# Unsupervised Clustering Subtype Analysis and Prognostic Risk Model of Cuproptosis-Related Genes for Liver Cancer

**DOI:** 10.5152/tjg.2025.24490

**Published:** 2025-08-11

**Authors:** WenKai Huang, QingSong Wu

**Affiliations:** 1 Department of Hepatobiliary Surgery, Yuebei People’s Hospital Affiliated to Shantou University, Guangdong, China

**Keywords:** Cuproptosis-related genes, hepatocellular carcinoma, prognostic risk model

## Abstract

**Background/Aims:**

To screen cuproptosis-related genes (CRGs) and construct a prognosis risk model for hepatocellular carcinoma (HCC) based on transcriptome data.

**Materials and Methods:**

Transcriptome, gene expression, and clinical data of HCC were downloaded from the Cancer Genome Atlas (TCGA) and Gene Expression Omnibus (GEO) databases to screen CRGs. Differential genes were screened, and Cox analysis and LASSO regression analysis were performed. The clinical value of the constructed model for HCC patients was assessed. Patient survival rates were predicted. The expression of relevant genes in liver cancer tissues and adjacent tissues was verified, and the prognostic risk for patients was evaluated.

**Results:**

Nineteen CRGs were identified, and 15 genes were expressed differently in tumor tissues and normal tissues. Multivariate analysis and LASSO regression analysis showed that 15 genes related to prognostic risk were screened, based on which a prediction model of 9 CRGs was constructed. High-risk patients, as determined by the prognostic model, showed a significantly decreased survival rate relative to low-risk patients. Tumor microenvironment and drug sensitivity were closely related to risk scores. Nomograms predicted survival probabilities for liver cancer patients over 1-, 3-, and 5-year periods at 91.6%, 62.4%, and 56.3%, respectively. Reverse transcription-quantitative polymerase chain reaction experiments verified the relevant gene expression that made up the model in liver cancer and adjacent tissues.

**Conclusion:**

The constructed prognostic risk model can predict the prognosis of HCC well and may be used for risk stratification, immunotherapy evaluation, and drug susceptibility analysis.

**Main Points**

- Nineteen cuproptosis-related genes were identified, and 15 genes were expressed differently in tumor tissues and normal tissues.
- Patient survival was significantly lower in the high-risk group, according to the prognostic model.
- Tumor microenvironment and drug sensitivity were closely related to risk scores.
- Reverse transcription-quantitative polymerase chain reaction experiments verified relevant gene expression that made up the model in liver cancer and adjacent tissues.

## Introduction

Liver cancer stands as the third most common cause of cancer-related deaths across the globe.^1^ Major risk factors for hepatocellular carcinoma (HCC) include hepatitis B virus (HBV) or hepatitis C virus (HCV) infection, chronic alcohol consumption, and diabetes. It is rare for surgical resection to cure early liver cancer in most patients.^2^ The opportunity for radical surgery is often lost because most liver cancer patients are diagnosed at an intermediate to advanced stage. Hepatocellular carcinoma is the predominant form of primary liver cancer, with approximately 830 000 new cases reported globally in 2020.^3^ An increasing number of combination therapies, such as immune checkpoint inhibitors in combination with tyrosine kinase inhibitors, are currently being tried, but the prognosis is still suboptimal. In spite of studies demonstrating major risk factors,^4^ the pathogenesis of HCC is not yet fully understood.^5^ As a highly heterogeneous malignant tumor, there is an urgent need to clarify the key factors involved in the development of HCC, explore its underlying mechanisms, and search for new and effective therapeutic targets.

Tumor development may be affected by copper imbalance through oxidative stress in the body.^6^ Copper targets lipidized tricarboxylate circulating proteins to induce cell death, which is associated with a variety of liver diseases. Copper ions bind directly to the lipoylated parts of the tricarboxylic acid cycle, creating proteotoxic stress that leads to cell death.^7^^,^^8^ Hepatocellular carcinoma cells show an increased synthesis of copper-binding proteins, which in turn leads to a significant increase in the copper content of the cell cytoplasm and a weakened ability to counteract the oxidative stress damage associated with copper overload.^9^ A cohort study showed a correlation between serum copper levels and the prognosis of HCC patients, with higher concentrations associated with a worse prognosis.^10^ Mitochondrial metabolic reprogramming is also one of the phenomena that often accompany copper imbalance, leading to excess ROS production and thus cellular oxidative stress.^11^ CuO nanoparticles can induce apoptosis of HCC HepG2 cells through reactive oxygen species (ROS) via the mitochondrial pathway.^12^ Copper-based therapies show the potential to inhibit tumor growth, especially for tumors that are insensitive to chemotherapy, and may provide new strategies for cancer treatment.^13^ However, there is still a need to investigate the mechanisms by which copper toxicity genes contribute to HCC.

Exploring the predictive significance of cuproptosis-related genes (CRGs) and their link to tumor mutations and immunotherapy involved gathering liver cancer specimens from The Cancer Genome Atlas (TCGA) and Integrated Gene Expression Omnibus (GEO) databases to thoroughly examine CRGs’ expression, mutation condition, and copy number differences. By analyzing the differential expression and prognostic analysis of CRGs, a new prognostic gene model was constructed and its efficacy was verified. Additionally, pathway analyses were performed to further evaluate the model’s value in molecular therapy by exploring the impact of risk scores on tumor mutation load and immunotherapy.

## Materials and Methods

### Research Workflow

The research workflow diagram for this study is shown in Supplementary Figure 1.

### Collection and Processing of Multi-Omics Data

By accessing the TCGA database (https://portal.gdc.cancer.gov/), data on gene transcriptomes (n = 424), clinical features (n = 377), and gene mutations (n = 364) from liver cancer patients were downloaded. Data in TPM format were extracted from this and normalized to log2 (TPM+1), and samples with RNA-Seq data and clinical information were ultimately retained. Next, through access to the GEO database (https://www.ncbi.nlm.nih.gov/geo/), the GSE76427 data sets were downloaded to obtain corresponding gene expression profile data and clinical data containing prognostic information. The relevant files from the TCGA data set were converted and processed using a Perl script to obtain liver cancer gene expression files and clinical data files, the samples with incomplete information were removed, and the data were normalized. The GEO database and TCGA data were merged to identify the intersection. The clinical features of all patients with HCC are shown in Table S1. Consensus analysis was performed using the “ConsensusClusterPlus” R package, and an unsupervised cluster analysis was performed on all samples from TCGA and GEO to identify patient subtypes based on prognosis-related differentially expressed genes (DEGs) . A heatmap of DEGs associated with prognostic CRGs was created using the “pheatmap” package, and to assess differences in gene expression between subtypes and produce box plots, the Kruskal–Wallis test was used.

Data on RNA sequencing, along with associated clinical and subsequent details, were sourced from the TCGA and GEO (GSE76427) databases. This research incorporated liver cancer patients for subsequent examination. Data on tumor node metastasis (TNM) stage, pathological grade, age, gender, duration of follow-up, and survival condition were collected. Nineteen regulatory genes associated with the metabolic pathway of copper toxicity^26^ have been identified, including NLRP3, NFE2L2, DLST, ATP7A, FDA1, DLD, LIPT1, PDHA1, DLAT, PDHB, GCSH, GLS, MTF1, DBT, and CDKN2A, as well as 4 negatively regulated genes, FDX1, LIAS, SLC31A1, and ATP7B (Table S2).

### Screening, Identification, and Prognostic Analysis of Cuproptosis-Related Genes

The RNA-Seq data of all samples were normalized using the R software’s limma package to analyze the differential expression of genes between normal tissues and tumor tissues. The intersecting genes were obtained by processing the gene data from the TCGA database with the “limma” and “sva” packages of R v4.3.0 (R foundation for Statistical Computing; Vienna, Austria). After excluding all normal samples, the remaining tumor samples were merged to create an integrated gene expression file for liver cancer tumor samples. Cuproptosis-related genes were screened using the R “limma” package to identify DEGs between normal and tumor tissues. *P*-values less than .05 were considered to be significantly different. The “maftools” and “RCircos” packages were used to analyze CRG mutations and copy number variation (CNV), and one-way Cox regression analysis was performed using the R language software package “survival” to screen the correlation between the previously obtained DEG levels and clinical prognosis.

### Model Construction and Verification

According to the random sampling method, all samples were divided into a train group, a test group, and an all group at a 1:1 ratio. For the training set data, one-factor significant gene expression matrices were analyzed using least absolute shrinkage and selection operator (LASSO) regression via the “glmnet” package in the R software, with a 10-fold cross-validation to select the optimal penalty coefficient (λ) for the LASSO significant gene expression data. A multifactorial Cox analysis of the genes significant in LASSO was performed using the R language software “survival” package to create a risk score model. Patient classification was based on the median risk score, verified through receiver operating characteristic (ROC) analysis. To evaluate the prognostic risk model’s effectiveness as an independent predictor of prognosis in HCC patients, both univariate and multivariate Cox regression analyses were conducted using the log-rank test, where a *P*-value below .05 indicated a statistically significant difference. The R software packages used here include timeROC, survival, and survminer. Overall survival (OS) results were compared using Kaplan–Meier analysis across all cohorts. In addition, prognostic differences between subgroups by clinical stage were analyzed.

### Constructing a Nomogram to Evaluate the Prognosis of Patients with Liver Cancer

Cox regression analyses were conducted on both univariate and multivariate data to identify possible independent prognostic factors. A nomogram was developed using the R “rms” package, taking into account risk scores and clinical factors such as gender, age, and tumor stage. Furthermore, calibration curves were generated for 1, 3, and 5-year OS in order to compare the model with actual performance. A consistency index (C-index) was also calculated using the C-index method to measure the alignment between the predicted outcomes and the actual observations. A higher C-index indicates a greater degree of accuracy for the predictive model.

### Collection and Processing of Tissue Samples

A total of 18 cases of cancer and their paired normal tissues were obtained from liver cancer patients undergoing surgery at the hospital. This study was approved by the Research Ethics Committee of Shantou University Affiliated Yuebei People‘s Hospital (Approval No. 202205GD93; approval date: May 23, 2022), and each patient provided written informed consent. Inclusion criteria were as follows: (1) Radical hepatectomy or hepatectomy for HCC and a clear postoperative pathological diagnosis; (2) primary tumor surgery and no prior radiotherapy, chemotherapy, immunotherapy, or other neoadjuvant therapy; (3) no previous tumor history; (4) complete clinicopathological data of patients; and (5) no obvious acute inflammatory diseases. Exclusion criteria were as follows: (1) Patients with tumors at other sites; (2) samples of small cancer tissue that would affect postoperative pathology; and (3) postoperative follow-up time of less than 1 month or loss to follow-up. The tissue sampling criteria were as follows: (1) Sampling should be completed within 30 minutes after isolation. At least 1 pair of liver cancer tissue and para-cancer tissue should be collected from each patient. The central part of the tumor should be removed, and the para-cancer tissue should be removed from the edge of the tumor above 3 cm; (2) after 2 washes with PBS, the necrotic tissue was removed, and the remaining tissue was cut into tissue blocks with a diameter of about 0.5 cm, which were respectively put into 1.5 mL EP tubes, immersed with RNALater, and stored in a refrigerator at −80°C; and (3) 18 liver cancer samples and 18 para-cancer samples were selected for reverse transcription-quantitative PCR (RT-qPCR) and collected at the same time.

### Reverse Transcription-Quantitative Polymerase Chain Reaction

Total RNA was extracted using Trizol reagent (ThermoFisher). cDNA synthesis corresponding to mRNA was carried out using the PrimeScript RT kit with gDNA Eraser (Takara) and SYBR Green qPCR Master Mix kit (Beyotime). Reverse transcription-quantitative polymerase chain reaction was performed with specific PCR primers. The internal reference gene was GAPDH. The primers are listed in Table 1.

### Statistical Analysis

Statistical analysis and data processing were performed using Perl software v5.30.0 (The Perl Foundation, Texas, USA) and R software v4.2.2 (R Foundation for Statistical Computing; Vienna, Austria). Continuous variables are indicated as mean ± SD. Reverse transcription-quantitative polymerase chain reaction was performed with specific primers. Cuproptosis-related genes (CRGs) and differentially expressed CRGs (DECRGs) were analyzed using Kruskal–Wallis rank sum testing. The clinicopathological features of the training set and the test set were analyzed using the chi-square test. Log-rank tests were employed to compare OS with median OS, while Wilcoxon tests were used to examine the relationship between characteristic genes and immune checkpoint expression. The Spearman technique was used for correlational studies, the Wilcoxon test for intergroup comparisons, Cox regression for assessing survival risk and hazard ratio (HR), LASSO regression for developing risk models, and Kaplan–Meier and log-rank tests for contrasting survival disparities among groups. A *P*-value below .05 was deemed to hold statistical significance.

## Results

### Screening, Identification, and Analysis of Prognostic Cuproptosis-Related Genes

**Figure 1.**
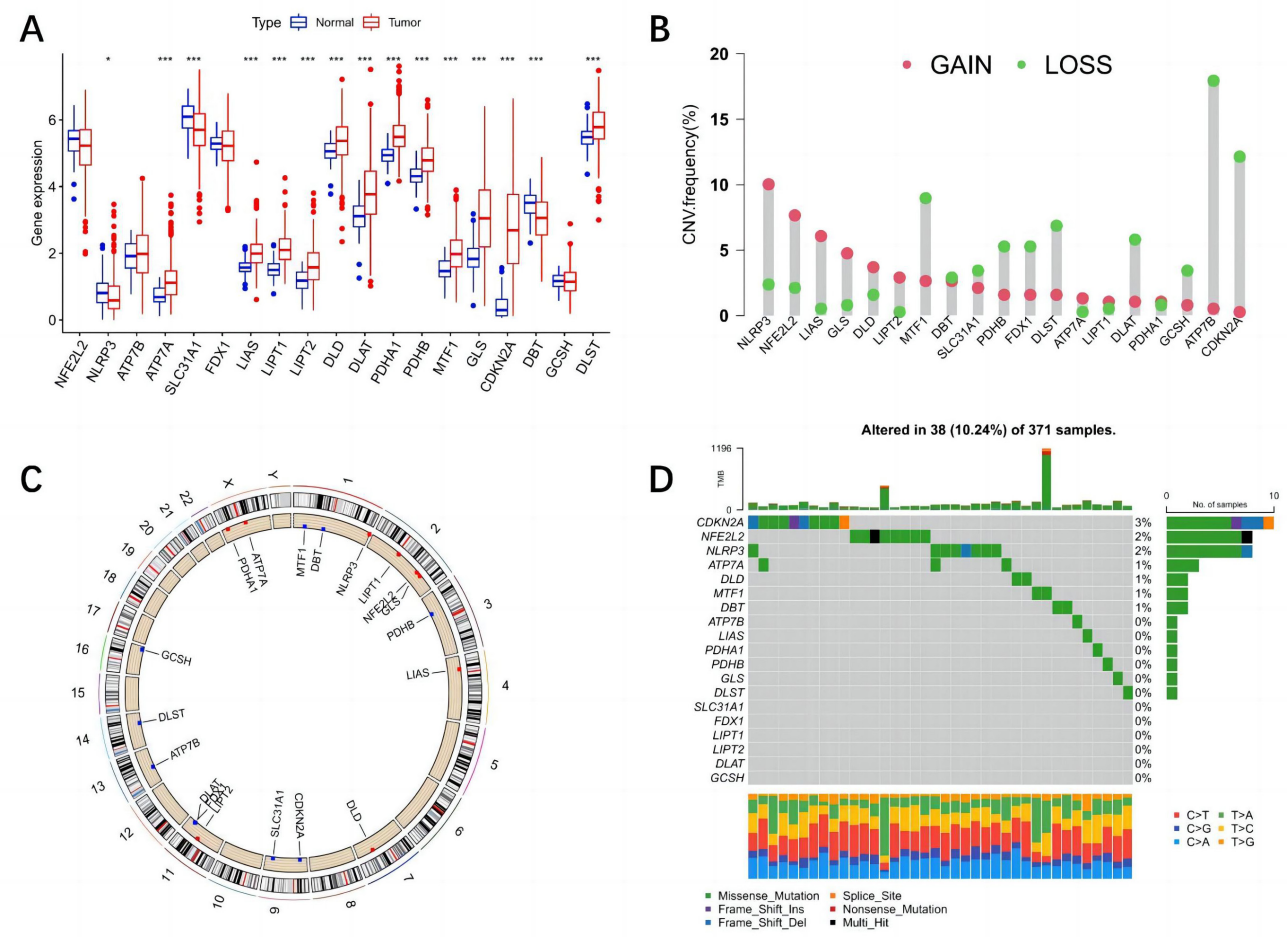
Expression and gene changes of CRGs in liver cancer. A: Distribution of differential expression of CRGs in tumor tissues and normal tissues in the TCGA dataset. B: Frequencies of CNV gain, loss, and non-CNV among CRGs. Red indicates copy number gain, and green indicates copy number loss. C: Circos plots of chromosome distribution of CRGs. Outer circles represent chromosomes; inner circles represent locations corresponding to CRGs; red is copy number gain of CRGs, blue is loss, and black is no significant gain or loss. D: Tumor mutation frequencies of CRGs. Horizontal coordinates represent HCC mutation load samples, vertical coordinates represent CRGs on the left side, while the right side represents the mutation frequencies of the corresponding genes; nonsense mutations are shown in red, missense mutations in green, splice sites in orange, deletions in yellow, multiple mutations in black, and no mutations in gray (*P < .05, **P < .01, ***P < .001). CNV, copy number variation; CRGs, cuproptosis-related genes.

Among the 19 CRGs included in this study, 15 were differentially expressed in normal tissue and tumor samples (Figure 1A), among which 6 were downregulated genes and 13 were upregulated genes. Among 371 liver cancer samples, 38 (10.24%) had CRG mutations (Figure 1B), 4 genes had CNV deletions, and 10 genes had CNV increases (Figure 1C). The CNVs of CRGs across 23 chromosomes were obtained (Figure 1D).

**Figure 2.**
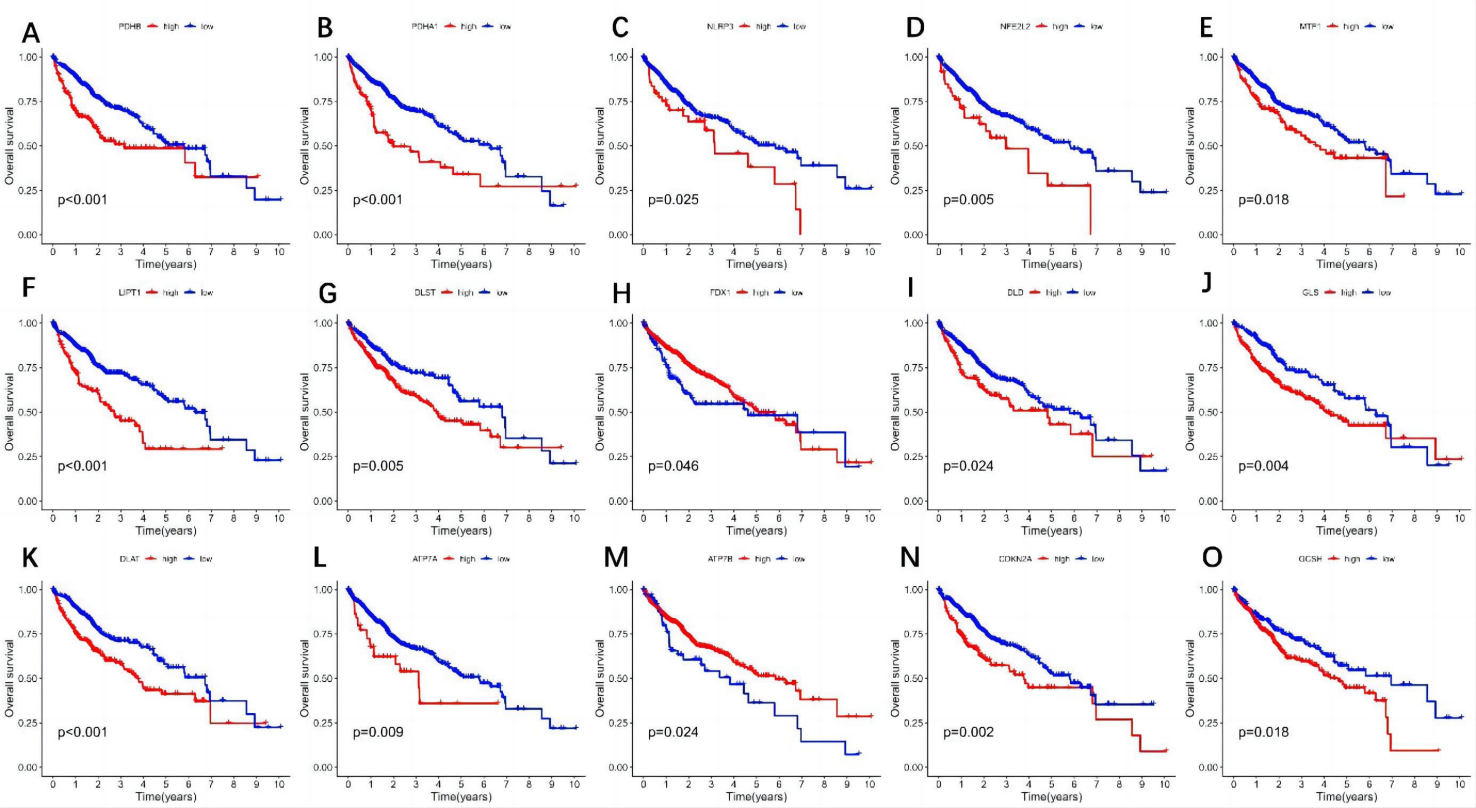
Prognostic significance of CRGs in patients with HCC. A-O: Kaplan–Meier analysis between 15 highly and lowly expressed CRGs with OS in HCC patients. CRGs, cuproptosis-related genes; HCC, Hepatocellular carcinoma; OS, overall survival.

Univariate Cox regression and Kaplan–Meier analyses showed 15 genes correlated significantly with liver cancer prognosis (*P* < .05) (Table 2). NLRP3, NFE2L2, DLST, ATP7A, DLD, LIPT1, PDHA1, DLAT, PDHB, GCSH, GLS, MTF1, DBT, and CDKN2A were high-risk prognostic genes (HR > 1, *P* < .05). FDX1, LIAS, SLC31A1, and ATP7B were protective genes (HR < 1, *P* < .05). The Kaplan–Meier curve illustrates that patients with HCC exhibiting elevated ATP7B and FDX1 gene levels experienced a prolonged OS in contrast to those with lower expression, whereas those with high expression levels had a reduced OS compared to their low expression counterparts (Figure 2).

### Prognostic Risk Model Based on Cuproptosis-Related Genes

**Figure 3.**
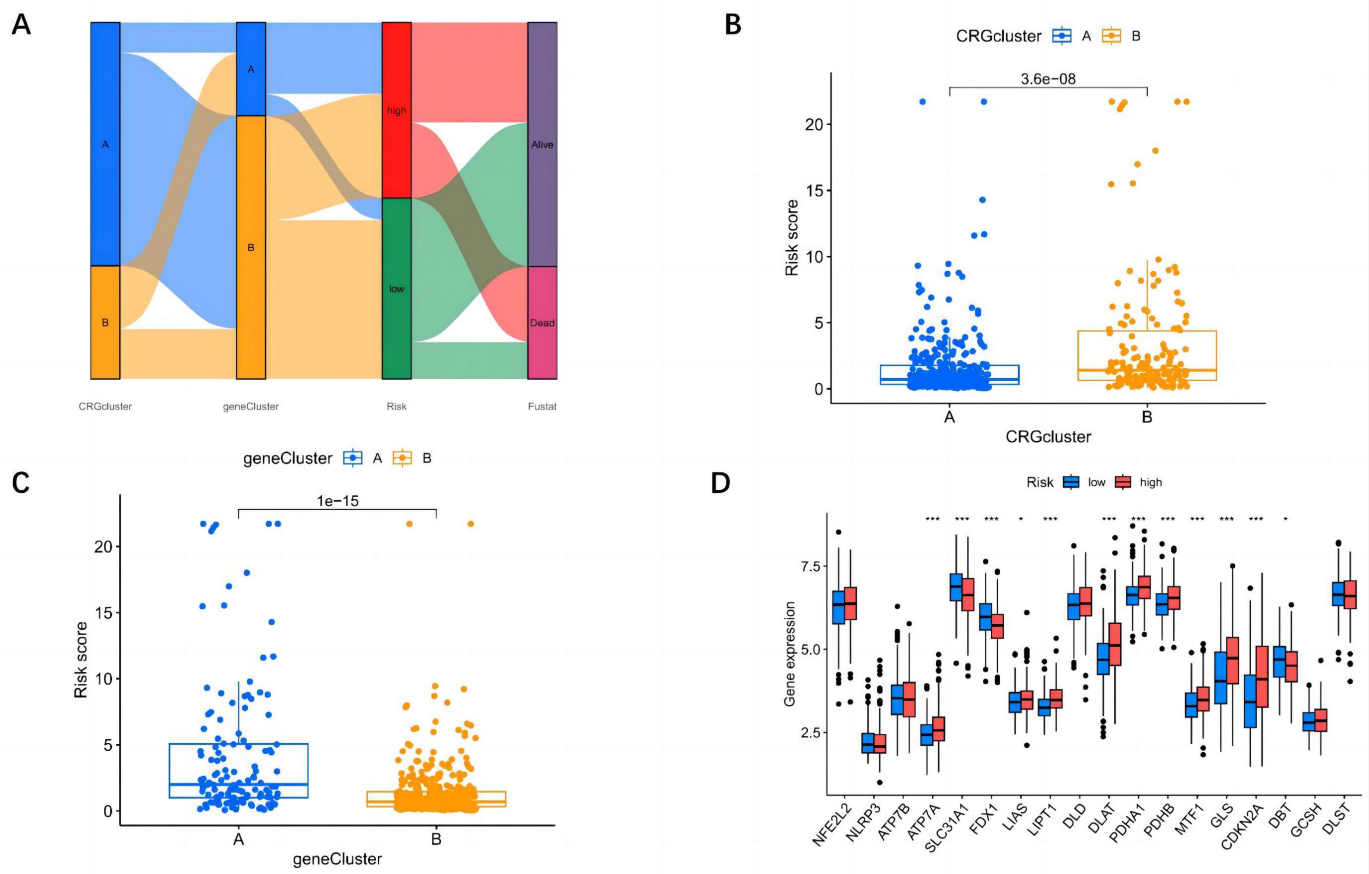
Model construction, model risk score relationship, and difference analysis of CRGs. A: Alluvial diagram of subgroup distributions in groups with different model risk scores and prognostic outcomes. B: Differences in risk_score between the 2 angiogenesis clusters. C: Differences in risk_score between the 2 gene clusters. D: Expression of CRGs in the high and low-risk groups. CRGs, cuproptosis-related genes.

Sankey’s chart was plotted to illustrate the distribution of subtypes, model risk scores, and prognosis from cluster analysis (Figure 3A). First, 487 patients were randomly divided into a train set (n = 244) and a test set (n = 243). Univariate Cox and LASSO regression analyses were performed on 15 DEGs. Nine prognostic risk genes (AGFG1, IQGAP3, MYO1E, FTCD, POF1B, IL7R, S100A9, CXCL9, and SPINK1) were screened out (Figure 3B-3D), and the prognostic model of the 9 genes was constructed with risk score = (0.8186 * AGFG1 expression) + (0.3142 * IQGAP3 expression) + (−0.6841 * MYO1E expression) + (−0.2103 * FTCD expression) + (0.1900 * POF1B expression) + (−0.2288 * IL7R expression) + (0.0903 * S100A9 expression) + (−0.1591 * CXCL9 expression) + (0.0787 * SPINK1 expression).

**Figure 4.**
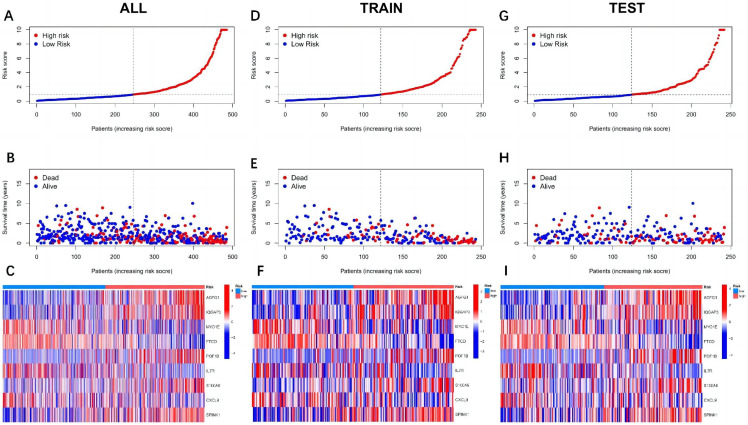
Risk curves for each group. A, B, and C are the all group; D, E, and F are the train group; and G, H, and I are the test group. The X-axis for each group is shared, representing the shared ascending risk score sample. A, D, G: Risk curves for high- and low-risk groups in the prognostic model (Y-axis is risk score). B, E, H: Scatterplot of patient survival in high- and low-risk groups in the prognostic model (Y-axis is survival time; blue represents survival, red represents death). From left to right, there are more and more red dots, fewer and fewer blue dots, and survival time is getting shorter and shorter. C, F, I: Risk heatmap of high- and low-risk groups in the prognostic model.

Patients were split into high-risk and low-risk groups according to the median risk score, with risk curves plotted for the train group, the test group, and all groups (Figure 4). The risk of death increased with the risk score in the train, test, and all groups. Meanwhile, the differential expression of CRGs between high and low-risk groups was observed, including 4 downregulated genes (MYO1E, FTCD, IL7R, and CXCL9) and 5 upregulated genes (AGFG1, IQGAP3, POF1B, S100A9, and SPINK1).

**Figure 5.**
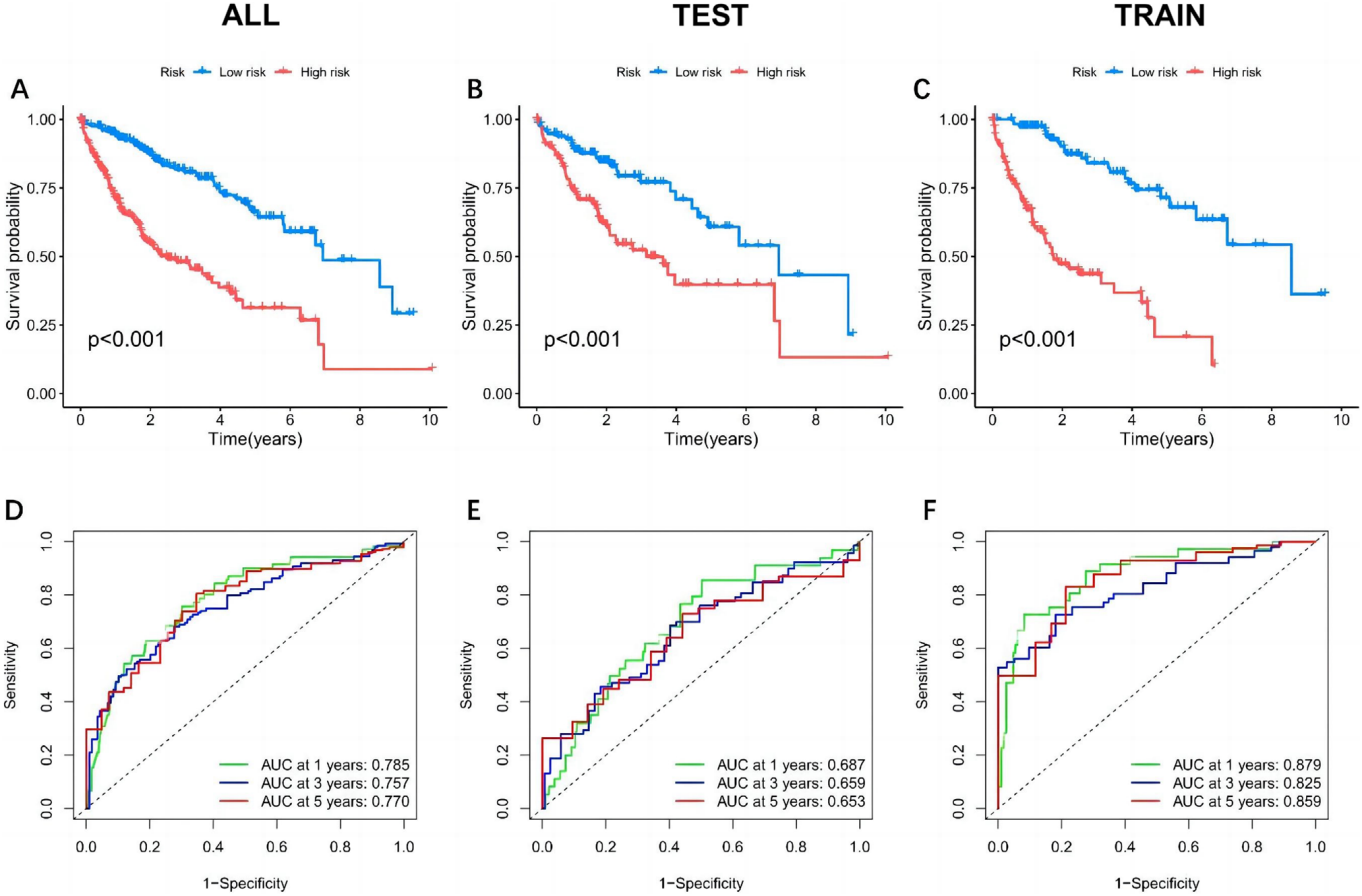
Model survival curve analysis and ROC curve. A and D are the all group, B and E are the test group, and C and F are the train group. A-C: survival analysis of high- and low-risk groups in the prognostic model. D: Survival prediction analysis with ROC curves according to the prognostic model.

**Figure 6.**
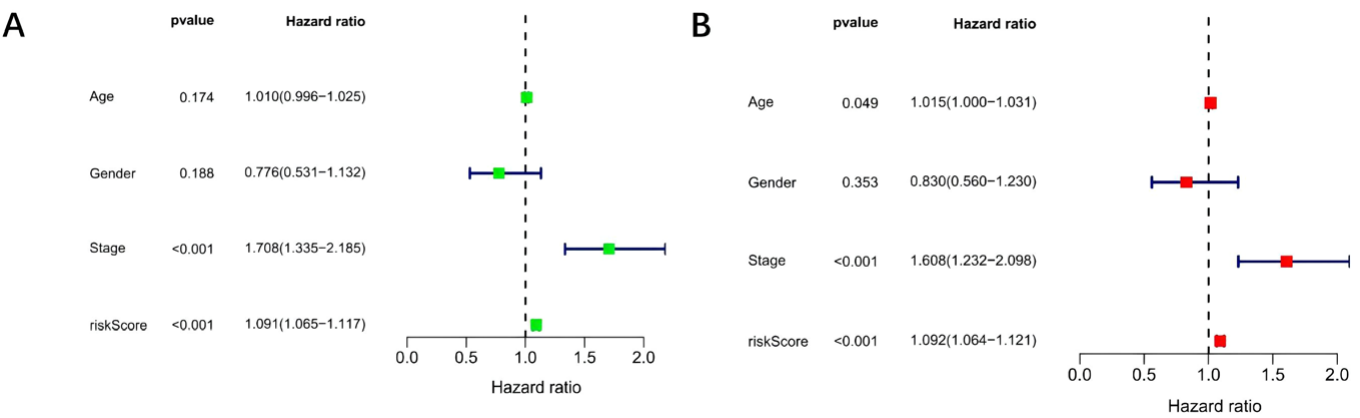
Assessment of the effect of forest maps and array maps in independent prognostic analysis. A: Univariate Cox analysis of factors influencing hepatocellular carcinoma prognosis. B: Multivariate Cox analysis of independent factors influencing hepatocellular carcinoma prognosis.

In the case of DEGs, patients were arbitrarily segregated into train, test, and all groups. The Kaplan–Meier analysis revealed that individuals in the high-risk group of the train group had a much shorter survival duration than those in the low-risk group (*P* < .05). Similar results were observed across the test and all groups (all *P* < .05) (Figure 5). The ROC analysis of the model was conducted using the “timeROC package” in R software, and ROC curves were generated for 1, 3, and 5 years. The 1-year, 3-year, and 5-year area under the curve (AUC) of the all groups were 0.785, 0.757, and 0.770, respectively, indicating that the prognostic risk model had good predictive performance (Figure 5). The model was validated using univariate and multivariate Cox regression analyses. According to the forest plot, OS was significantly correlated with risk score and clinical stage, whereas gender and age could not be used as independent factors to determine prognosis (Figure 6).

### Predictive Value of the Model

**Figure 7.**
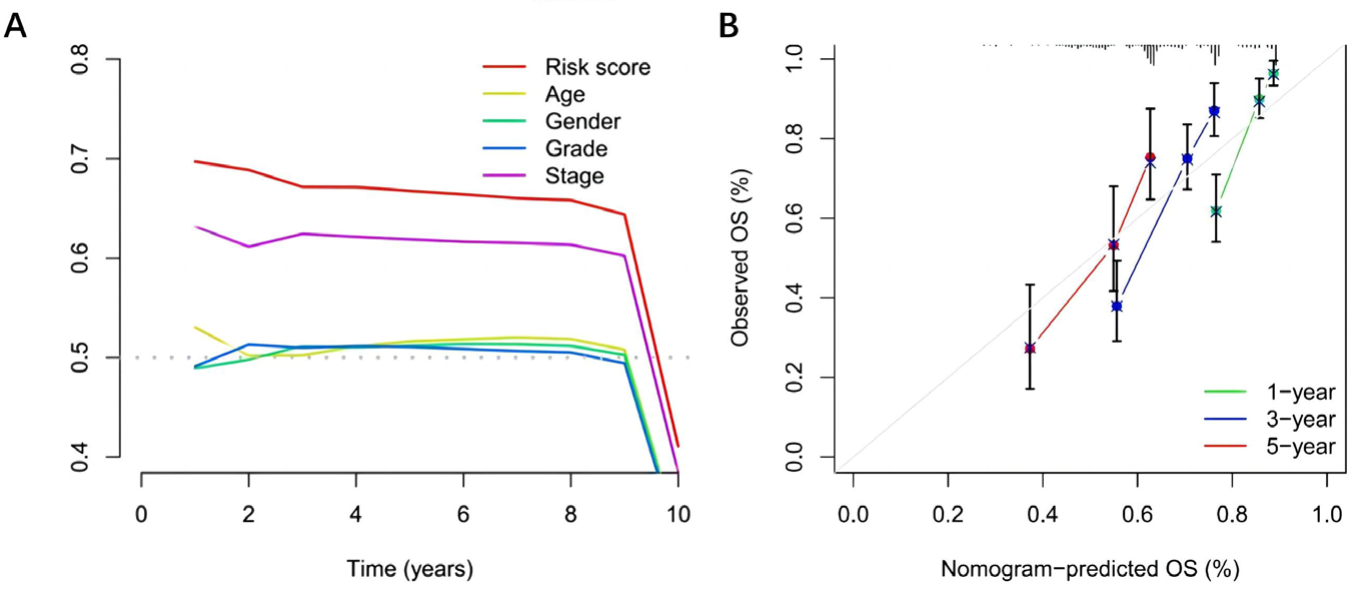
Assessment of the effect of forest maps and nomograms in independent prognostic analyses. A: Nomogram for predicting the 1-, 3-, and 5-year overall survival of hepatocellular carcinoma patients in the entire cohort. B: C-Index curves.

**Figure 8.**
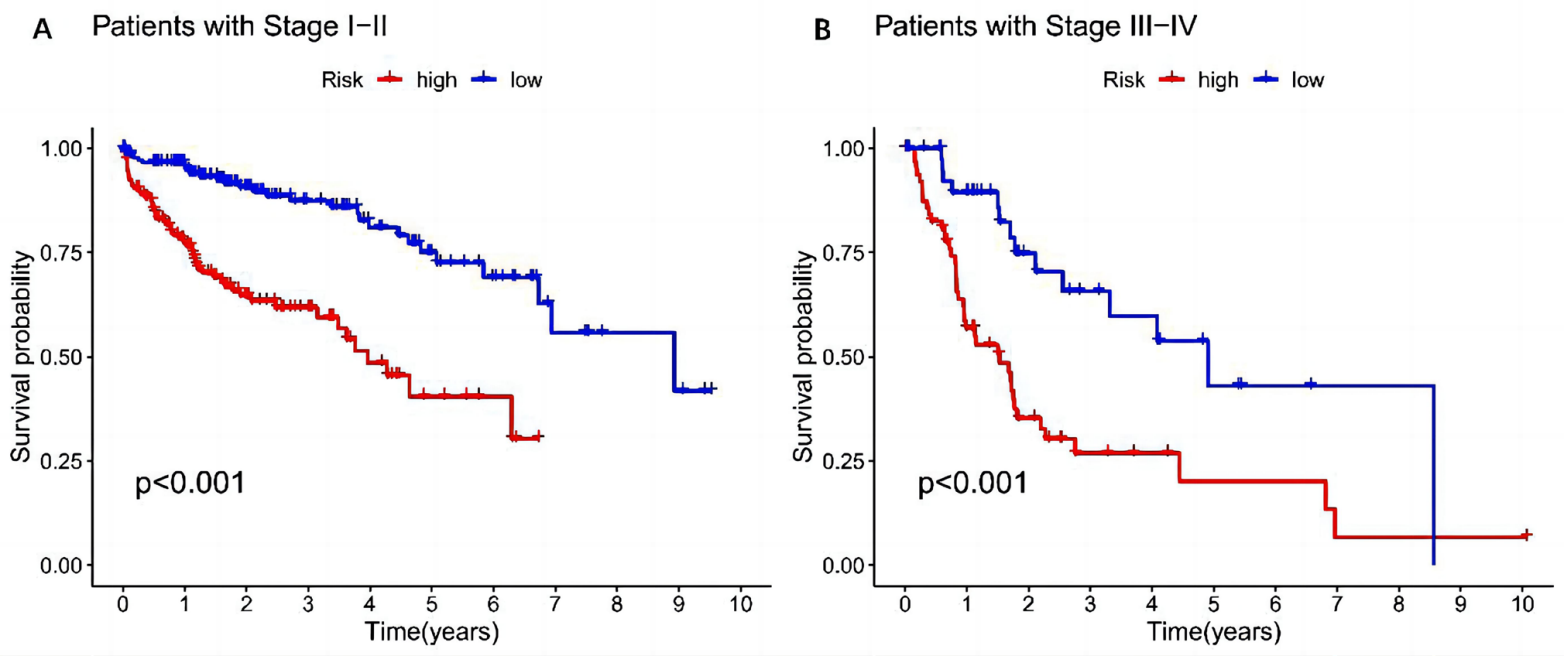
Survival curves of high-risk and low-risk patients with different tumor stages. A: Tumor stage I to II. B: Tumor stage III to IV.

A total score of 122 points was obtained by considering the contributions of different factors to the survival rate. The rates for 1-year, 3-year, and 5-year survival were 93.1%, 84.8%, and 74.2%, respectively. The nomogram’s calibration curve demonstrated superior predictive ability for 1-year, 3-year, and 5-year survival compared to the ideal model (Figure 7A). C-index analysis showed that the C-index of the model was 0.700, indicating that the nomogram had a relatively good prognostic value (Figure 7B). Finally, a clinical stratification analysis was performed to determine clinical factors (tumor stage). Results demonstrated a marked improvement in survival rates for low-risk patients with stage I-II or stage III-IV tumors compared to high-risk patients (*P* < .001) (Figure 8).

### Differential Expression Analysis and Prognosis Analysis of Model Genes

**Figure 9.**
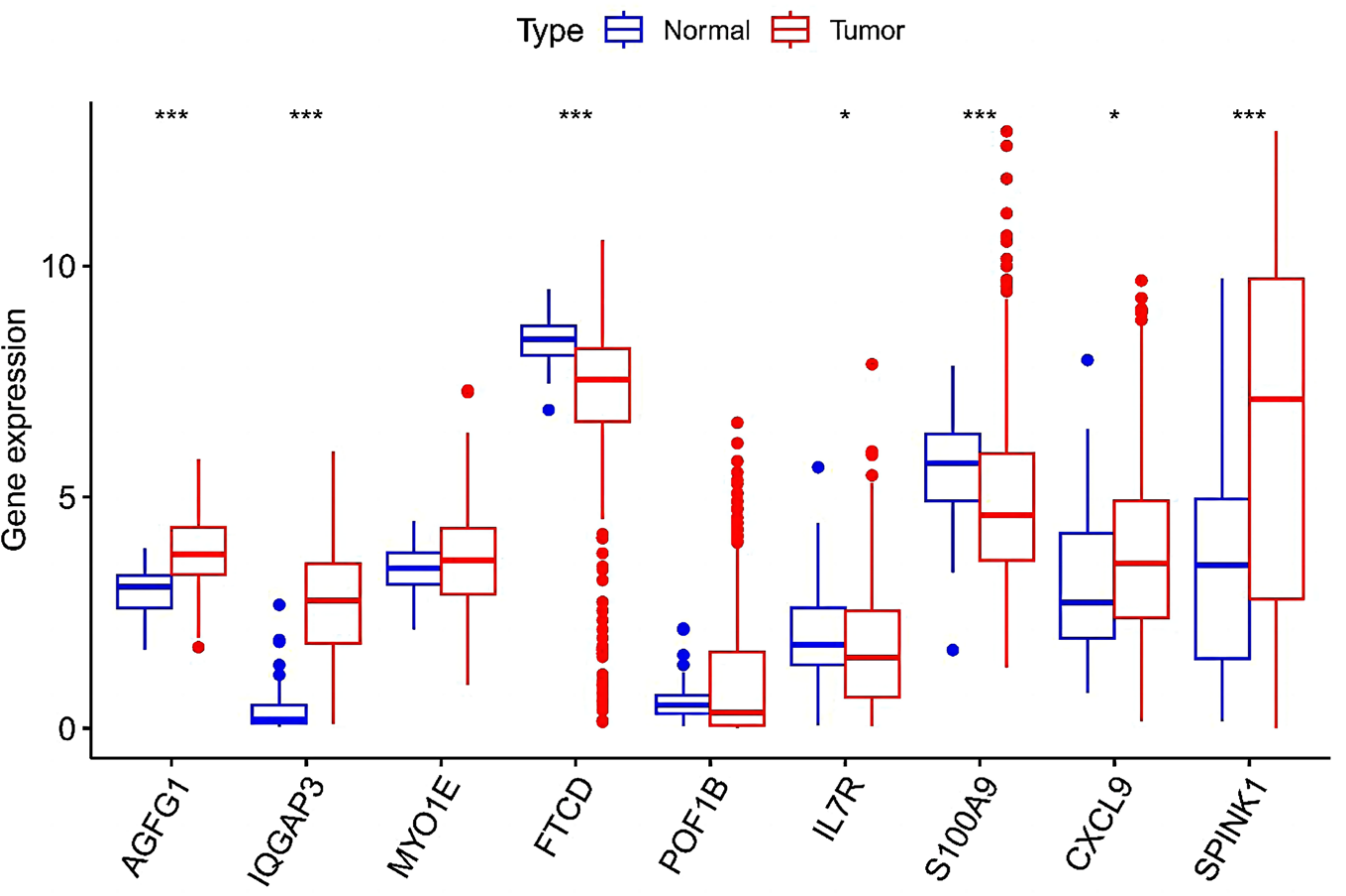
Differential expression of model genes in tumor tissues and normal tissues in the Cancer Genome Atlas database.

**Figure 10.**
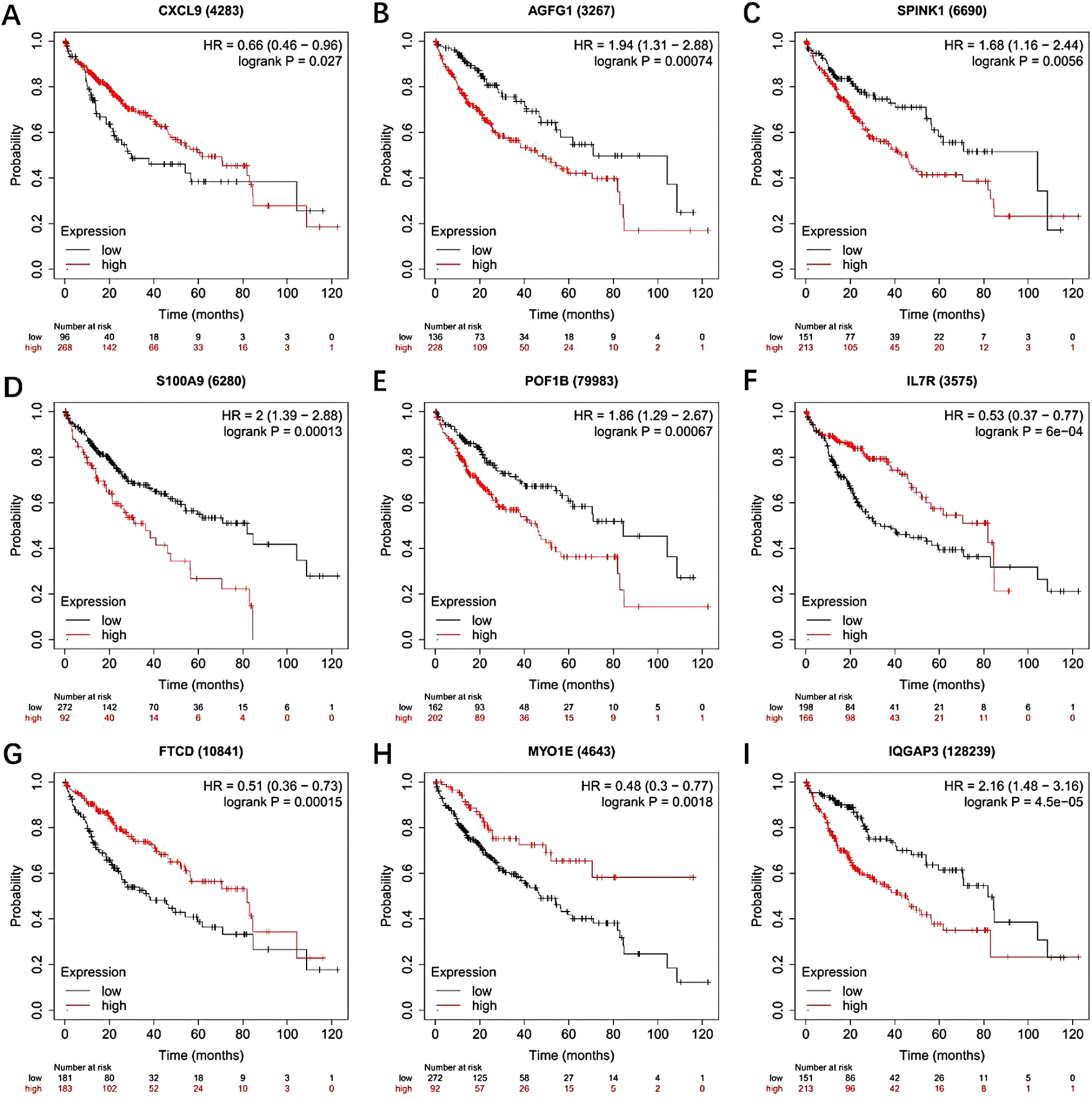
Survival curve analysis of genes associated with hepatocellular carcinoma prognosis at high and low expression levels. A-I: Survival curve analysis of 9 genes associated with the hepatocellular carcinoma prognosis model at high and low expression.

The reliability of the model was further explored by conducting a differential analysis of gene expression on TCGA data. The analysis of TCGA liver cancer data showed that the expressions of AGFG1, IQGAP3, SPINK1, CXCL9, MYO1E, and POF1B were significantly increased in tumor tissues (Figure 9). The expressions of S100A9, FTCD, and IL7R were increased in normal tissues. The Kaplan–Meier Plotter (http://kmplot.com/analysis/index.php?p=service) was used for analyzing model genes and OS of HCC patients. Hepatocellular carcinoma patients’ prognosis was significantly impacted by all 9 genes, according to the KM curve (Figure 10).

### Expression of Prognostic Genes in Liver Cancer and Normal Tissues

**Figure 11.**
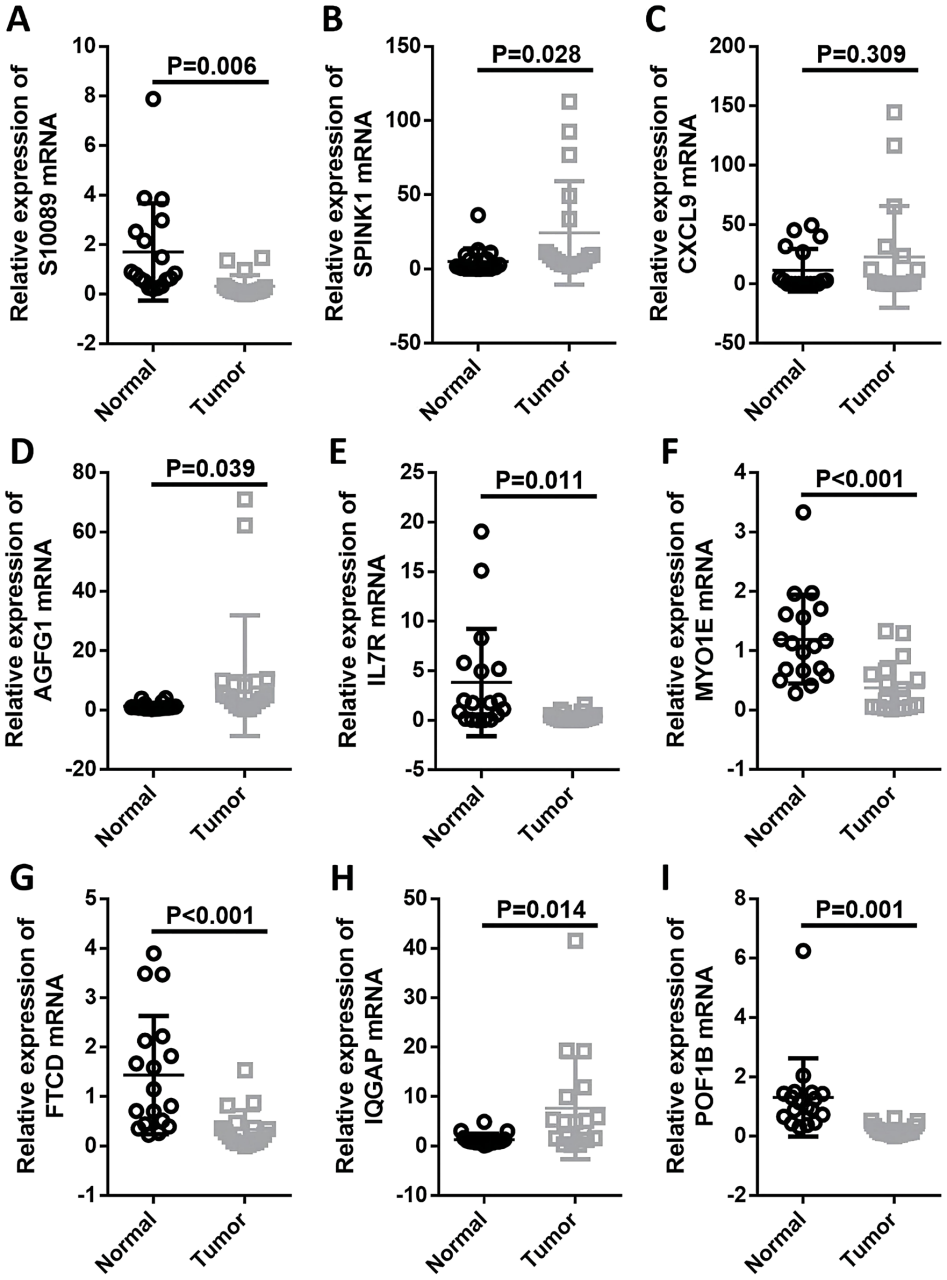
Reverse transcription-quantitative polymerase chain reaction detection of mRNA expression of hepatocellular carcinoma prognosis-related genes in tumor tissues and normal tissues. A-I: Expression levels of 9 genes associated with the hepatocellular carcinoma prognosis model in tumor tissues and normal tissues.

Through bioinformatics analysis, a prognostic model of liver cancer associated with 9 DECRGs was obtained. Normal tissues exhibited higher levels of S100A9, FTCD, POF1B, IL7R, and MYO1E, whereas tumor tissues showed increased expressions of SPINK1, CXCL9, AGFG1, and IQGAP3 (Figure 11).

## Discussion

There will be more than 1 million cases of liver cancer in 2025, a steadily rising rate.^14^ Cuproptosis, a new form of programmed cell death, exhibits unique characteristics that set it apart from oxidative stress-related cell death, which is positively associated with cancer progression.

Through the application of univariate regression, LASSO Cox regression, and multifactor regression analyses, 9 DECRGs were obtained. Following this, 9 DECRGs were employed to create a predictive risk model, assessed through survival, mutational, and independent prognostic analyses, demonstrating strong predictive capabilities.

It has been reported that some model genes are associated with the prognosis and progression of liver cancer and other tumors. AGGF1 is a common oncogene with high levels within tumors such as glioblastoma, colorectal, and gastric cancers. Notably, AGGF1 can promote angiogenesis of liver cancer, is overexpressed in liver cancer, and predicts poor prognosis.^15^ In addition to this, overexpression of AGGF1 significantly promotes HCC progression by rescue experiments.^16^ These results are in agreement with this study, but no further exploration in animal experiments was pursued. Xi et al^17^ found that HBV induced S100A9 to activate RAGE/TLR4-ROS signals, which promoted the growth and metastasis of HCC.^18^ This study’s results are consistent with previous findings that S100A9 is related to poor prognosis in HCC patients. Man et al^20^ found that SPINK1 knockout can inhibit the growth of liver cancer and its ability to resist chemotherapy.^19^ FTCD shows a high level of expression in the liver, yet its expression is markedly reduced in HCC. Regularly, the use of FTCD immunostaining, either singly or alongside other proteins, has demonstrated considerable diagnostic importance in the initial detection of HCC. Low levels of FTCD in HCC patients are associated with poor prognoses, while cirrhosis and low levels of FTCD are associated with a significantly higher risk of HCC.^20^ Hepatocellular carcinoma tumors that express high levels of CXCL3 are associated with a worse prognosis compared to those with low CXCL3 expression. In addition, Zhang et al^22^ showed that CXCL3 played a key role in humoral immune infiltration in the development of cirrhosis-related liver cancer.^21^ At present, there is no study on the relationship between MYO1E and liver cancer, but the study by Liu et al^23^ showed that MYO1E is mainly expressed in pancreatic adenocarcinoma (PAAD) and is negatively correlated with the survival and prognosis of patients.^22^ This is in line with the survival curve analysis outcomes for varying MYO1E expression, suggesting that MYO1E may serve as a useful therapeutic target in HCC. POF1B is mainly expressed in polarized epithelial tissues, and the aberrant expression of POF1B is closely related to malignant tumors such as cutaneous squamous cell carcinoma and lung adenocarcinoma. Fourteen genes, including POF1B, have previously been combined to form a prognostic model for predicting HCC prognosis with vascular infiltration,^23^ which implies that POF1B could be a novel biomarker for HCC prognosis. However, at present, the potential mechanisms linking the prognosis of HCC patients with these 9 genes remain unclear, which can be the focus of future research.

A significant difference in feature genes was found between neighboring non-tumor samples and HCC samples from the TCGA gene database and the GSE76427 dataset. Similar results were obtained by conducting qRT-PCR analysis. In the GEPIA2 survival data, these genes showed significant associations with prognosis. A regression coefficient suggests that IQGAP3 is the most important DECRG in terms of prognostic factors and prognostic prediction. IQGAP3 functions as a scaffolding protein, interacting with diverse structural proteins to alter cytoskeletal dynamics and intracellular signaling, thus influencing the proliferation and migration of tumors.^24^ IQGAP3 expression correlates with aggressive clinicopathologic features.^25^ Moreover, IQGAP3 promotes Epithelial-Mesenchymal Transition (EMT) and metastasis through activation of the Ras/ERK pathway and transforming growth factor and intracellular 26^,27^ and activation of the Wnt/EMT, and metastasis through activation of resistance in HCC cells. Various previous findings support the inclusion of IQGAP3 as a prognostic model for HCC in this study. Moreover, the role of IQGAP3 in the regulation of downstream targets and pathways in HCC needs to be deeply explored.

The study constructed a prognostic prediction model for HCC consisting of 9 CRGs based on the TCGA database and the GEO database, which had good predictive accuracy, differentiation, and clinical efficacy. The validation demonstrated that the model could help identify high-risk populations, stratify disease risk management, and provide personalized prevention programs and that the predictive information obtained through the model could prevent the further development of poor prognosis of HCC.

This research, however, is subject to certain limitations. The data of this model is retrospective, lacking comprehensive verification from external datasets. Additionally, the sample size was small, despite partial expression testing at the tissue level. A future study will assess the relevance of the model for immunotherapy and examine how immunotherapy differs between low-risk and high-risk patients. Second, this study lacked a stratified analysis of the model in different subgroups. The model cannot be clinically assessed for its applicability to patients of different ages, genders, and stages of tumors. At the same time, the lack of studies on the robustness of the model also affects the judgment and treatment decision-making for patients with complex clinical characteristics, which not only affects the promotion of the model in clinical practice but also reduces the acceptance and utility of the model in the clinic. This is a problem that needs to be overcome and further explored in the future.

In summary, a novel prognostic CRG model has been developed to enhance the prediction of HCC prognosis, aiding clinicians in evaluating patient outcomes and informing treatment strategy development.

## Supplementary Materials

Supplementary Material

## Figures and Tables

**Table 1.** Primer Sequence

Gene	Forward primer (5’ →3’)	Reverse primer (5’ →3’)
S100A9	CAGCTGAGCTTCGAGGAGGTT	CGTGCATCTTCTCGTGGGAG
FTCD	ATGTCCCAGCTGGTGGAATG	CCAGGTACTCGATGATCCGC
POF1B	CTCTGCACACTGGCAACAAC	CCGCAACTTGGAGAGTTCCT
IL7R	AAATATGTGGGGCCCTCGTG	AAGTCATTGGCTCCTTCCCG
MYO1E	GCAAGTGCAGTTCCACCAAG	TCCTTCTGGTAGGACGGGAG
SPINK1	TGACTCCCTGGGAAGAGAGG	AGTCCCACAGACAGGGTCAT
CXCL9	TGAGAAAGGGTCGCTGTTCC	GGGCTTGGGGCAAATTGTTT
AGFG1	GGTGGTGATCAGGGAAGTGG	ACCAGCAGCAGCAACAAATG
IQGAP3	TGGGATTGGCCCCTCAGATA	AGCTCCTTCACAGTGTCAGC
GAPDH	GTCAAGGCTGAGAACGGGAA	AAATGAGCCCCAGCCTTCTC

**Table 2.** Analysis of Survival Prognosis of Cuproptosis-Related Genes

Genes	HR (95% CI)	*P* (Cox)	*P* (KM)
NFE2L2 NLRP3 ATP7B ATP7A SLC31A1 FDX1 LIAS LIPT1 DLD DLAT PDHA1 PDHB MTF1 GLS CDKN2A DBT GCSH DLST	1.244(1.010-1.532) 1.302(0.957-1.770) 0.939(0.764-1.156) 1.409(1.051-1.890) 0.969(0.783-1.200) 0.988(0.767-1.273) 0.993(0.694-1.420) 2.042(1.401-2.977) 1.154(0.908-1.467) 1.318(1.107-1.570) 1.322(1.001-1.745) 1.422(1.046-1.934) 1.330(0.999-1.772) 1.199(1.044-1.379) 1.152(1.017-1.305) 1.015(0.791-1.304) 1.385(0.969-1.979) 1.098(0.845-1.426)	.0400.040 .093 .553 .022 .775 .927 .967 .001 .242 .002 .049 .025 .051 .010 .026 .905 .074 .484	.005 .025 .024 .009 .065 .046 .080 6.66E-06 .024 .001 6.45E-05 .001 .018 .004 .002 .163 .018 .005

## Data Availability

The data that support the findings of this study are available on request from the corresponding author.
