# Supplementary material for: Unsupervised Clustering Subtype Analysis and Prognostic Risk Model of Cuproptosis-Related Genes for Liver Cancer
Source: Turk J Gastroenterol. 2025 Aug 11;37(1):75–87. doi: 10.5152/tjg.2025.24490 (PMC12824887; doi:10.5152/tjg.2025.24490)
Supplement: Supplementary Material [file supplementary_material.pdf]

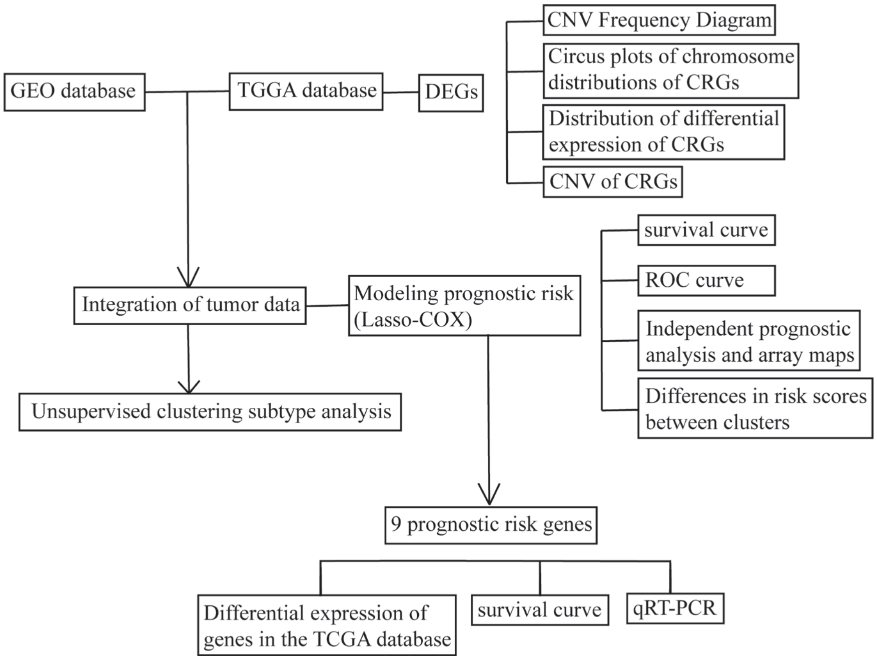

Supplementary Figure 1. The workflow diagram for bioinformatics research.

Supplementary Table 1. Analysis of clinical information data based on GEO database and TCGA data

Variables	TCGA cohort (N = 376)	GSE76427 cohort (N = 115)
Age		
≤ 65 years	235	65
>65 years	141	50
Gender		
Male	122	22
Female	254	93
Classification		
G1	55	NA
G2	180	NA
G3	123	NA
G4	13	NA
Unknown	5	NA
Staging		
I	175	55
II	86	35
III	86	31
IV	5	3
Unknown	24	1
T classification		
T1	185	NA
T2	94	NA
T3	81	NA
T4	13	NA
TX	1	NA
Unknown	2	NA
M classification		
M0	272	NA
M1	4	NA
MX	100	NA
N classification		
N0	257	NA
N1	4	NA
N2	114	NA
Unknown	1	NA
Overall survival		
Dead	132	23
Survived	244	92

Cancer Genome Atlas (TCGA).
TCGA and Gene Expression Omnibus (GEO) databases (GSE76427) databases.

Supplementary Table 2. Abbreviations

Genes	Name
NFE2L2	Nuclear factor, erythroid 2 like 2
NLRP3	NACHT, LRR, and PYD domains-containing protein 3
ATP7B	P-type ATPase gene
ATP7A	Copper transporter copper-transporting ATPase 1
SLC31A1	Solute carrier family 31 member 1
FDX1	Ferredoxin 1
LIAS	Lioyl synthase
LIPT1	Lipoyltransferase 1
LIPT2	Lipoyltransferase 2
DLD	Dihydrolipoamide dehydrogenase
DLAT	dihydrolipoamide S-acetyltransferase
PDHA1	Pyruvate dehydrogenase E1 component subunit alpha
PDHB	Pyruvate dehydrogenase beta subunit
MTF1	Metal response element binding transcription factor 1
GLS	Glutaminase
CDKN2A	cyclin-dependent kinase inhibitor 2a
DBT	Dihydrolipoamide branched chain transacylase E2
GCSH	Glycine Cleavage System Protein H
DLST	dihydrolipoamide S-succinyltransferase
